# Glycemic variability is associated with vascular calcification by the markers of endoplasmic reticulum stress-related apoptosis, Wnt1, galectin-3 and BMP-2

**DOI:** 10.1186/s13098-019-0464-4

**Published:** 2019-08-19

**Authors:** Li Zhang, Haichen Sun, Shuang Liu, Jinhuan Gao, Jinggang Xia

**Affiliations:** 1 Department of Geriatrics, Xuanwu Hospital, Capital Medical University, China National Clinical Research Center for Geriatric Medicine, Beijing, 100053 China; 2 Surgical Laboratory, Xuanwu Hospital, Capital Medical University, Beijing, 100053 China; 3 Department of Cardiology, Xuanwu Hospital, Capital Medical University, Beijing, 100053 China

**Keywords:** Glycemic variability, Calcification score, Markers

## Abstract

**Background:**

The present study identified whether glycemic variability (GV) was associated with vascular calcification and explored the underlying mechanisms.

**Methods:**

Eighty-four consecutive type 2 diabetic patients with unstable angina (UA) were included from January 2018 to June 2018 to calculate calcification scores using computerized tomographic angiography (CTA), and the patients were divided into 2 groups: high calcification score group (HCS group) and low calcification score group (LCS group). Intergroup differences in GV were determined via comparisons of the standard deviation (SD) of blood glucose. Calcification staining, content measurement, apoptosis evaluation and Western blot analysis of endoplasmic reticulum (ER) stress-related apoptosis, Wnt1, galectin-3 and bone morphogenetic protein-2 (BMP-2) were compared in cell cultures from rat vascular smooth muscle cells in the different degrees of GV.

**Results:**

The SD increased significantly with the increases in calcification scores from human studies (HCS group 2.37 ± 0.82 vs. LCS group 1.87 ± 0.78, *p *= 0.007). Multivariate logistic regression analysis suggested that increased SD and serum creatinine were independent predictors of calcification. The high GV group had a higher apoptotic rate, higher calcification content and higher expressions of glucose-regulated protein, caspase-3, Wnt1, galectin-3 and BMP-2 markers compared to the low GV group in the in vitro studies (*p *< 0.001).

**Conclusion:**

We report the novel finding that GV is associated with vascular calcification, and ER stress-related apoptosis, Wnt1, galectin-3 and BMP-2 may be involved in this regulation.

## Introduction

Reducing adverse events from diabetic-induced cardiovascular disease (CVD) remains an imperative clinical challenge [1]. Calcification is a special manifestation of CVD that induces major arterial stiffening and occlusions. Severe coronary artery calcification increases the risks of interventional procedures, and patients have a poor prognosis [2]. Diabetes mellitus is a primary cause of vascular calcification [3]. The mechanism through which diabetes mellitus promotes vascular calcification is not clear. Recent studies have shown that glycemic variability (GV) is more dangerous than persistent hyperglycemia and that GV is a marker of progression of CVD [4, 5]. However, whether GV is associated with vascular calcification is not clear, and the possible mechanisms remain elusive. The related clinical and basic data are lacking. The present study examined the association between GV and calcification and the possible mechanisms using cell culture.

## Methods

### Human studies

#### Study population

This study was a prospective observational study of GV and vascular calcification in type 2 diabetic patients with unstable angina (UA) from January 2018 to June 2018 at the Department of Cardiology, Xuanwu Hospital. The Ethics Committee of Xuanwu Hospital approved the clinical and animal study protocols, which were performed in accordance with The Code of Ethics of the World Medical Association (Declaration of Helsinki). Consecutive patients with UA complicated with type 2 diabetes mellitus (T2DM) were screened for eligibility. All patients gave written informed consent. UA was defined as ischemic chest pain or aggravation occurring within 1 month before admission without elevation of troponin.

We excluded patients with any of the following characteristics: (1) previous coronary artery bypass graft; (2) contrast agent allergy; (3) a glomerular filtration rate (eGFR) < 30 mL/min/1.73 m^2^; (4) cardiac function classification (NYHA) ≥ 3.

#### Study design

A total of 84 patients were included and had calcification scores calculated using computerized tomographic angiography (CTA). Four coronary arteries (the left anterior descending, left circumflex, right coronary artery and left main) were analyzed by Agatston score quantification. The patients were divided into 2 groups: a high calcification score group (HCS group) (≥ 100, n = 31) and a low calcification score group (< 100, LCS group) (n = 53). All patients received conservative treatment, interventional therapy and coronary artery bypass therapy based on the doctor’s decision. Demographic data, medical history/risk factors and blood samples were collected.

#### Measurement of GV

Fingertip blood samples for fasting blood glucose (FBG) and postprandial blood glucose (PBG) (2 h after breakfast, lunch and dinner) were measured. The means and SD of all blood glucose values during hospitalization for each patient were calculated. The times of the FBG and PBG measurements depended on the days of hospitalization.

### In vitro studies

#### Cell culture, identification and grouping

The animal studies followed the Guide for the Care and Use of Laboratory Animals published by the US National Institutes of Health (NIH Publication No. 85-23, revised 1996). Vascular smooth muscle cells (VSMCs) were isolated and cultured from the abdominal aorta of 4-week-old male Sprague–Dawley rats according to the previous Ref. [6]. The purity of VSMC cultures was confirmed using immunohistochemical staining with a specific α-smooth muscle actin antibody (α-SMA actin). Primary VSMCs at passages 3–8 were used in subsequent experiments. The identified VSMCs were divided into the following four groups according to the different media used for culture: (1) control group (C group), routine DMEM cultured for 10 days; (2) continuous hyperglycemia group (CH group), 25 mmol/L sugar DMEM cultured for 10 days; (3) low GV group (LGV group), 5 mol/L sugar DMEM and routine DMEM cultured alternately every 12 h for 10 days; and (4) high GV group (HGV group), 25 mol/L sugar DMEM and routine DMEM cultured alternately every 12 h for 10 days.

#### Calcium content measurement of VSMCs

VSMC calcification was visualized by von Kossa staining. The calcium content of VSMCs was measured with the Calcium Colorimetric Assay Kit according to the manufacturer’s instructions [7].

#### Apoptosis assay

Apoptosis was measured using the Annexin V-FITC Apoptosis Detection Kit (BD Pharmingen, San Diego, CA, USA). The samples were analyzed with a FACSCalibur flow cytometer (BD Biosciences San Diego, CA, USA).

#### Western blot assay of related markers

Cell homogenates and SDS-polyacrylamide gels for electrophoresis were prepared as described previously [8]. The membranes were incubated with different primary antibodies [GRP78 (1:1000) (Abcam, UK), caspase-3 (1:1000) (Cell Signaling Technology, USA), Wnt1 (1:1000) (Abcam, UK), galectin-3 (1:1000) (Cell Signaling Technology, USA), BMP-2 (1:1000) (Abcam, UK), and β-actin (1:2000) (Anhui, China)] overnight at 4 °C and visualized with the corresponding secondary antibody at room temperature for 1 h. Protein bands were visualized using enhanced chemiluminescence (ECL) (Millipore, Billerica, MA, USA), and the results were quantified using Image-Pro Plus 6.0 software and normalized to β-actin. The experiment was repeated three times.

### Statistical analysis

Student’s t-test was used to compare two variables, and one-way analysis of variance (ANOVA) was used to compare more than two groups. Proportions were compared using Fisher’s exact test when the expected frequency was less than five, and Chi square test was used otherwise. Logistic regression was used to determine the odds ratios and the 95% confidence intervals that assessed the association between GV and calcification according to potential confounding variables. To adjust for baseline differences, potentially relevant variables were included in models if they had univariable differences with p values < 0.05.

Results are expressed as the mean ± SD unless otherwise specified. All calculations were performed using SPSS 13.0, and *p* values < 0.05 (two-tailed) were considered significant.

The authors had full access to and take full responsibility for the integrity of the data. All authors have read and agreed to the manuscript as written.

## Results

### Human studies

A total of 84 patients were included during the study period. The mean age was 62 ± 11 years. The proportion of males was 67.9%. The HCS and LCS groups were similar in current smoker status, stroke history, history of dyslipidemia, sex, age, previous percutaneous coronary intervention, old myocardial infarction, proportion of patients with two vessel lesions, left ventricular ejection fraction, left ventricular end diastolic diameter, glycated hemoglobin A1c (GHbA1c), and serum lipids (*p *> 0.05).

More patients had a history of hypertension in the HCS group than the LCS group (*p *< 0.05). The HCS group had a greater proportion of patients with three vessel lesions and a lower proportion of patients with one vessel lesion than the LCS group (*p *< 0.05). The HCS group had higher blood glucose and serum creatinine values and SD at admission than the LCS group (*p *< 0.05). The HCS group had higher Global Registry of Acute Coronary Events (GRACE) risk scores and a greater proportion of patients with GRACE risk scores ≥ 140 at admission than the LCS group (*p *< 0.05). Baseline clinical factors/characteristics are shown in Table 1.

**Table 1:** Baseline characteristics/clinical factors

Variable	HCS group (n = 31)	LCS group (n = 53)	*p* Value
Sex, M/F	21/10	36/17	0.82
Age, years	63 ± 10	61 ± 11	0.39
History of hypertension	24	28	0.04
History of dyslipidemia	15	25	0.91
History of stroke	5	10	0.98
Current smoker	18	28	0.81
Previous MI	9	13	0.84
Previous PCI	5	8	0.85
No. diseased vessels			
LM	5	5	0.57
1	7	25	0.04
2	10	18	0.94
3	14	10	0.02
LVEDD, mm (before discharge)	48 ± 7	47 ± 9	0.57
LVEF, % (before discharge)	52 ± 9	51 ± 10	0.64
Blood sugar at admission, mmol/L	8.9 ± 1.5	8.1 ± 1.8	0.03
GHbA1c, %	7.4 ± 0.8	7.2 ± 1.2	0.36
Serum creatinine at admission, μmol/L	117.2 ± 29.9	101.4 ± 26.3	0.01
Total cholesterol, mmol/L	5.85 ± 1.72	5.79 ± 1.96	0.88
LDL-C, mmol/L	3.73 ± 0.69	3.69 ± 0.85	0.81
HDL-C, mmol/L	1.19 ± 0.55	1.23 ± 0.49	0.74
GRACE risk score in-hospital	129 ± 37	112 ± 38	0.04
GRACE risk score ≥ 140	16	14	0.04
Means of glucose values during hospitalization	10.3 ± 1.2	9.9 ± 1.5	0.21
Glycemic variability (SD) mmol/L	2.37 ± 0.82	1.87 ± 0.78	0.007

Values are given as n (%) or mean ± SD

*HCS* high calcification score, *LCS* low calcification score, *M* male, *F* female, *MI* myocardial infarction, *PCI* percutaneous coronary intervention, *LM* left main, *LVEDD* left ventricular end diastolic diameter, *LVEF* left ventricular ejection fraction, *GHbA1c* glycated hemoglobin A1c, *LDL-C* low-density lipoprotein cholesterol, *HDL-C* high-density lipoprotein cholesterol, *GRACE* Global Registry of Acute Coronary Events, *SD* standard deviation

Multivariate logistic regression analysis suggested that increased SD [odds ratio 2.57, (1.95–4.35), *p *= 0.01] and serum creatinine [odds ratio 2.95, (2.27–4.89), *p *= 0.02] were independent predictors of calcification.

### In vitro studies

#### The effect of GV on VSMC calcium

**Fig. 1:**
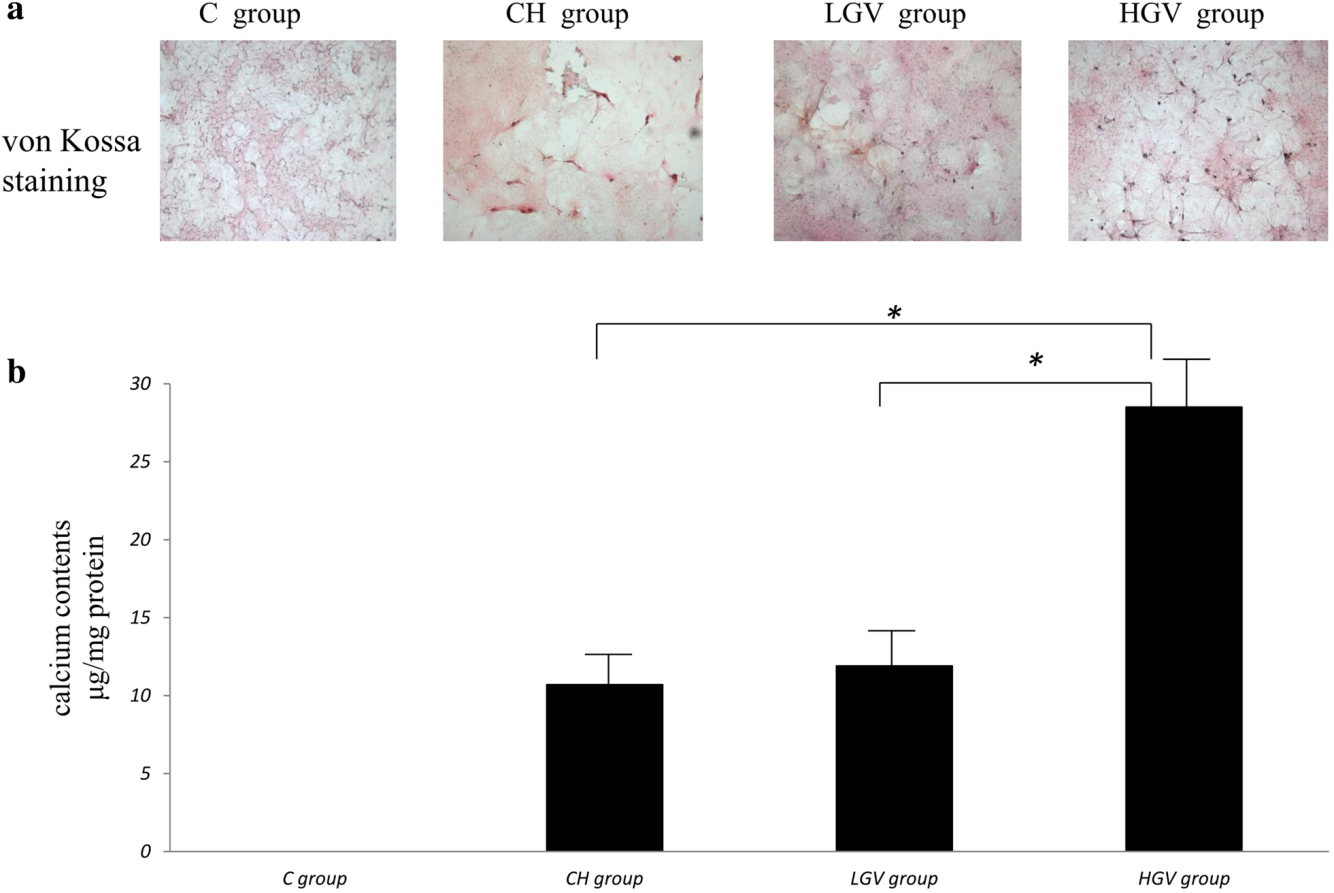
The effect of glycemic variability (GV) on calcium in vascular smooth muscle cells (VSMCs). Rat VSMCs were cultured in different medium according to GV for 10 days. **a** VSMC calcification was examined by von Kossa staining. **b** Calcium content was examined by the ocresolphthalein complex one method and was normalized to the cellular protein concentration. The calcium content was higher in the HGV group than the LGV and CH groups (*p *< 0.01). The calcium content was similar between the CH and LGV groups (*p *= 0.71) The data shown are representative of the results from three independent experiments, each performed in duplicate. *p < 0.05 compared with the individual groups

Calcification in any glucose treatment group (CH group, LGV group and HGV group) was higher than the C group using von Kossa staining. The HGV group exhibited higher calcification compared to the CH group and LGV group (Fig. 1a). The calcium content was higher in the HGV group than the LGV group (28.5 ± 4.2 μg/mg protein in HGV vs. 11.9 ± 3.9 protein μg/mg in LGV, *p *= 0.01) and the CH group (28.5 ± 4.2 μg/mg protein in HGV vs. 10.7 ± 3.5 μg/mg protein in CH, *p *= 0.005). The calcium content was similar between the CH and LGV groups (*p *= 0.71) (Fig. 1b).

#### Apoptosis assay

**Fig. 2:**
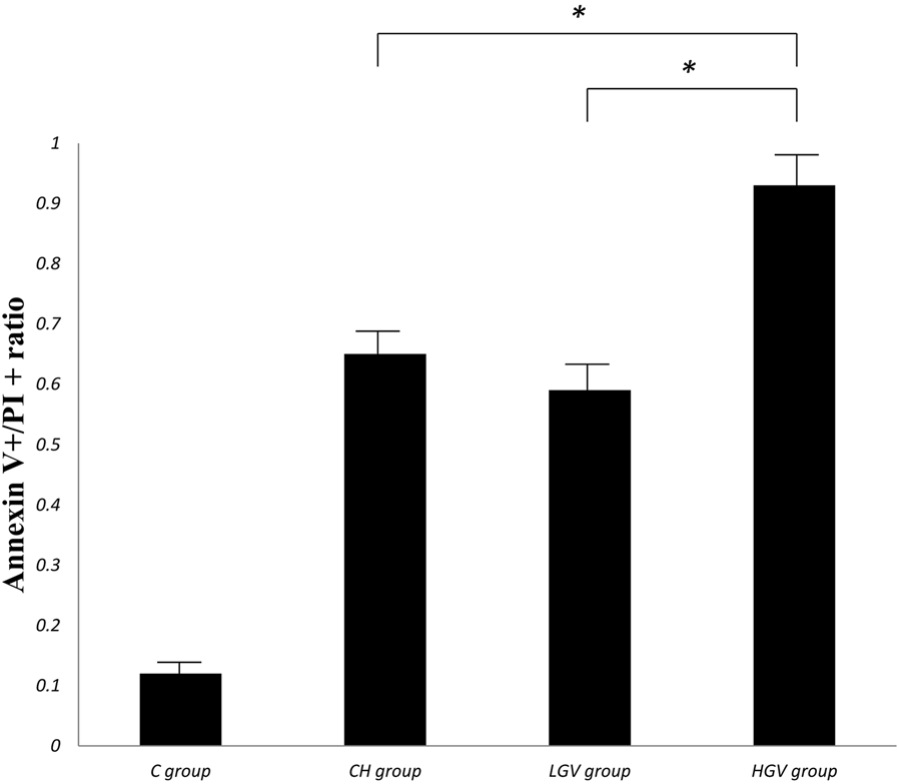
The relative ratio of the apoptotic cells (Annexin V positive/PI positive) was examined by flow cytometry. The data shown are representative of the results from three independent experiments, each performed in duplicate. *p < 0.05 compared with the individual groups

The relative ratio of apoptotic cells was determined using flow cytometry. The apoptotic rate in any glucose treatment group [CH group (0.65 ± 0.11) and the LGV group (0.59 ± 0.13) and the HGV group (0.93 ± 0.12)] was greatly increased compared to the C group (0.12 ± 0.09) (*p *< 0.05). The apoptotic rate in the HGV group was significantly greater than the CH group (*p *< 0.05) and the LGV group (*p *< 0.001). The apoptotic rate was similar between the CH group and LGV group (*p* > 0.05) (Fig. 2).

#### Endoplasmic reticulum (ER) stress-related apoptosis, Wnt1, galectin-3 and BMP-2 markers evaluated by protein levels

**Fig. 3:**
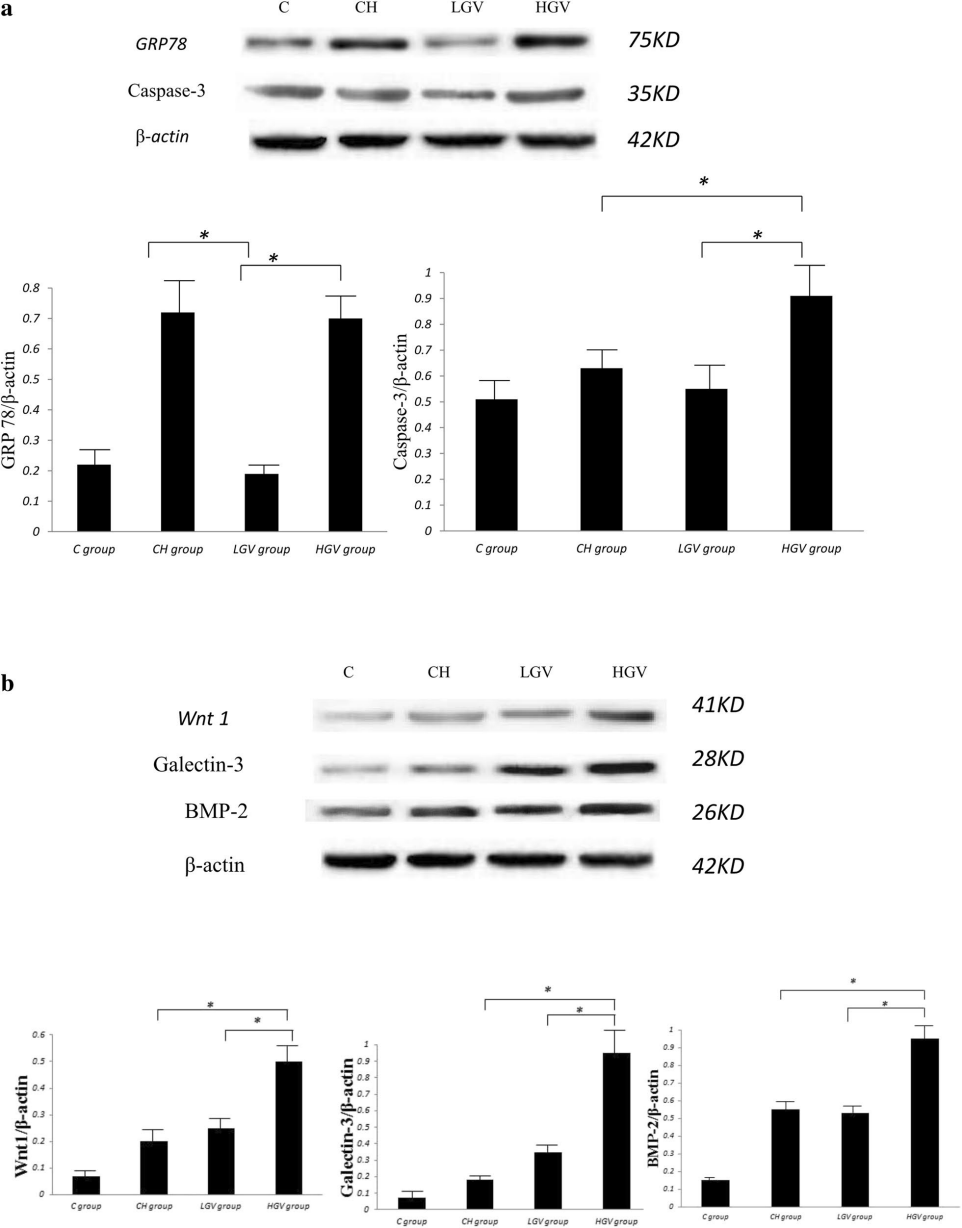
The protein expression of endoplasmic reticulum stress-related apoptosis, Wnt 1, galectin-3 and BMP-2 markers. **a** The protein expression of GRP78 and caspase-3 in different groups, and **b** the protein expression of the Wnt1, galectin-3 and BMP-2 in different groups. The intensity of individual protein bands obtained from Western blot was quantified by densitometry. β-actin was used as an internal loading control. Representative results from three independent experiments are shown. *p < 0.05 compared with the individual groups

The GRP78 protein expression in the HGV and CH groups was significantly higher than the LGV group (*p *< 0.001). The GRP78 protein expression was similar between the HGV and CH groups (*p* > 0.05). We also found that the caspase-3 protein expression in the HGV group was significantly higher than the CH group (p < 0.001) and the LGV group (p < 0.001). The caspase-3 protein expression was similar between the CH and LGV groups (*p* > 0.05) (Fig. 3a).

The Wnt1 protein expression in the HGV group was significantly higher than the CH group (*p *< 0.001) and the LGV group (*p *< 0.001). Wnt1 protein expression was similar between the CH and LGV groups (*p* > 0.05). The galectin-3 protein expression in the HGV group was significantly higher than the CH group (*p *< 0.001) and the LGV group (*p *< 0.001). The galectin-3 protein expression was similar between the CH and LGV groups (*p* > 0.05). The BMP-2 protein expression in the HGV group was significantly higher than the CH group (*p *< 0.001) and the LGV group (*p *< 0.001). BMP-2 protein expression was similar between the CH and LGV groups (*p* > 0.05) (Fig. 3b).

## Discussion

Our study explored the relationship between GV and vascular calcification and the underlying mechanisms. We found that (1) higher calcification scores correlated with higher GV, and (2) higher GV was related to a higher apoptotic rate, a higher calcification content, and higher expressions of GRP78, caspase-3, Wnt1, galectin-3 and BMP-2 markers. Our report is the first study to show the relationship between GV and vascular calcification and examine the possible pathways and mechanisms through which diabetes mellitus promotes calcification.

In human studies, we found that higher calcification scores were correlated with higher GV. Snell-Bergeon et al. performed a similar study [9]. They explored coronary artery calcium and its relation to GV in patients with type 1 diabetes and found that GV was consistently related to coronary artery calcification in men. They included patients with a mean age of 42 ± 9 years and type 1 diabetes duration of 29 ± 8 years. Our study included patients with T2DM with a mean age of 62 ± 11 years. We did not perform a subgroup gender analysis. We found that the HCS group had higher serum creatinine and SD than the LCS group. Multivariate logistic regression analysis suggested that the increased SD and serum creatinine were independent predictors of calcification. A previous study supported our results. Avogaro et al. [10] and Badin et al. [11] showed that diabetes was an important predisposing factor for vascular calcification. Chronic kidney disease often complicates long-standing diabetes, and this condition is associated with accelerated calcification [12]. We also found that the HCS group with higher GV had a greater proportion of patients with three vessel lesions, higher GRACE risk scores and an increased proportion of patients with GRACE risk scores ≥ 140 at admission than the LCS group, but the two groups had similar GHbA1c. Patients with similar GHbA1c values may have dramatically different GV and different degrees of vascular calcification, vascular lesion and risk for adverse events, which suggests that GV may be one of the ways in which diabetes induces vascular diseases independently.

To confirm whether GV exerted an independent effect on vascular calcification and the possible underlying mechanisms, we performed an in vitro study. We found that the HGV group had increased calcification content compared to the CH and LGV groups. These results suggest that the degree of GV is closely related to vascular calcification, and higher GV is more harmful than chronic, sustained hyperglycemia [4, 5].

We performed a preliminary study of the possible mechanisms and found that the apoptotic rate and the protein expressions of GRP78 and caspase-3 were higher in the HGV group than the LGV group. The ER regulates protein synthesis, lipid synthesis and calcium homeostasis. Endoplasmic reticulum stress (ERS) is a cellular response caused by homeostatic imbalance in the endoplasmic reticulum (ER). ERS was related to the progression of vascular calcification. High glucose in diabetics interferes with ER function, and ERS generates several proapoptotic signals and initiates apoptosis [13, 14]. The apoptosis of VSMCs that is caused by ERS promotes vascular calcification and the phenotype transformation of VSMCs [15, 16]. Notably, we found that the protein expression of GRP78 was similar between the HGV group and CH group, but the apoptotic rate and the protein expression of caspase-3 in the HGV group was significantly higher than the CH group. Moderate ERS increases the release of GRP78, which is involved in the recognition and degradation of misfolded proteins and is protective. However, severe stress causes apoptosis via increased activation of caspases [17]. Therefore, our result suggests that the higher GV that leads to excessive ER stress and activates more apoptosis is more harmful than chronic, sustained hyperglycemia and lower GV. Our result also indicates that the endoplasmic reticulum stress apoptotic pathway may be involved in the pathogenesis of diabetic vascular calcification.

We found that higher GV was related to higher protein expressions of Wnt1, galectin-3 and BMP-2 markers. The Wnt pathway causes vascular calcification via stimulation of calcification and regulates key aspects of diabetic vascular disease [18–21]. A previous study showed that galectin-3 was a potential biomarker of diabetic vasculopathy, and it was involved in endothelial dysfunction that leads to the vascular complications of T2DM [22]. Galectin-3 is also the key regulator of osteogenic differentiation, and it participates in the process of endochondral bone formation and is essential for a complete transdifferentiation of VSMCs into osteoblast-like cells via direct modulation of Wnt signaling [23–25]. BMP-2 is expressed and secreted from various cell types, including vascular smooth muscle cells and osteoblasts, and it induces vascular calcification and accelerates phosphate uptake [26]. Zhang et al. demonstrated that patients suffering from T2DM had higher levels of BMP-2 than normal controls. BMP-2 was positively correlated with calcification in patients suffering from T2DM [27]. The relationship between endoplasmic reticulum stress and the Wnt pathway was reported in tumors and cardiovascular research. Dr. Rodvold [28] reported that transmissible ER stress activated Wnt signaling in recipient cancer cells and enhanced resistance to nutrient starvation and common chemotherapies. Dr. Shen [29] demonstrated the pivotal role of reactive oxygen species in mediating ERS and functional impairment of cardiomyocytes via the C/EBP homologous protein-Wnt pathway.

This study had some limitations. First, it was a small study at a single-center. Second, research on more biomarkers may be more thorough at the in vitro level. Third, the application of positive and negative feedback in vitro signaling pathway research would be helpful for clarifying the mechanisms. Fourth, a single estimation of GV during hospital admission following an acute event may be not reflective of the patient’s GV at home in a stable condition and is not adequate to determine the association between vascular calcification and GV. In future studies, we will study the correlation between long-term GV using continuous glucose monitoring (CGM) and the incremental value of vascular calcification scores after discharge to further clarify the relationship.

## Conclusions

The present trial showed that GV was associated with vascular calcification, and ER stress-related apoptosis, Wnt1, galectin-3 and BMP-2 markers may be involved in this regulation. GV may become a new target for evaluation of calcification, cardiovascular risk, treatment strategy and prognosis in clinical practice.

## Data Availability

The datasets used and/or analysed during the current study are available from the corresponding author on reasonable request.

## Abbreviations

GV: glycemic variability
UA: unstable angina
CTA: computerized tomographic angiography
HCS: group high calcification score group
LCS: group low calcification score group
SD: standard deviation
ER: endoplasmic reticulum
BMP-2: bone morphogenetic protein-2
VSMCs: vascular smooth muscle cells
HGV: group high GV group
GRP78: glucose-regulated protein
LGV: group low GV group
CVD: cardiovascular disease
T2DM: type 2 diabetes mellitus
LM: left main
LAD: left anterior descending
LCX: left circumflex
RCA: right coronary artery
PCI: percutaneous coronary intervention
CABG: cardiac artery bypass grafting
FBG: fasting blood glucose
PBG: postprandial blood glucose
CH: continuous hyperglycemia group
ECL: enhanced chemiluminescence
MI: myocardial infarction
PCI: percutaneous coronary intervention
LVEDD: left ventricular end diastolic diameter
LVEF: left ventricular ejection fraction
GHbA1c: glycated hemoglobin A1c
GRACE: Global Registry of Acute Coronary Events
ERS: endoplasmic reticulum stress
